# Transplantation of Human Embryonic Stem Cell-Derived Retinal Pigment Epithelial Cells in Macular Degeneration

**DOI:** 10.1016/j.ophtha.2018.04.037

**Published:** 2018-11

**Authors:** Manjit S. Mehat, Venki Sundaram, Caterina Ripamonti, Anthony G. Robson, Alexander J. Smith, Shyamanga Borooah, Martha Robinson, Adam N. Rosenthal, William Innes, Richard G. Weleber, Richard W.J. Lee, Michael Crossland, Gary S. Rubin, Baljean Dhillon, David H.W. Steel, Eddy Anglade, Robert P. Lanza, Robin R. Ali, Michel Michaelides, James W.B. Bainbridge

**Affiliations:** 1 NIHR Biomedical Research Centre for Ophthalmology, Moorfields Eye Hospital and University College London, London, United Kingdom; 2 Moorfields Eye Hospital NHS Foundation Trust, London, United Kingdom; 3 Institute of Ophthalmology, University College London, London, United Kingdom; 4 Cambridge Research Systems Ltd., Rochester, United Kingdom; 5 University of Edinburgh, Edinburgh, United Kingdom; 6 University College Hospital, London, United Kingdom; 7 Newcastle Eye Centre, Newcastle upon Tyne, United Kingdom; 8 Casey Eye Institute, University of Oregon Health & Science University, Portland, Oregon; 9 Institute of Genetic Medicine, Newcastle University, Newcastle upon Tyne, United Kingdom; 10 Astellas Institute for Regenerative Medicine, Marlborough, Massachusetts; 11 Kellogg Eye Center, University of Michigan, Ann Arbor, Michigan

**Keywords:** BCVA, best-corrected visual acuity, FAF, fundus autofluorescence, hESC, human embryonic stem cell, STGD1, Stargardt disease, RPE, retinal pigment epithelial, SD, spectral-domain, VA, visual acuity

## Abstract

**Purpose:**

Transplantation of human embryonic stem cell (hESC)-derived retinal pigment epithelial (RPE) cells offers the potential for benefit in macular degeneration. Previous trials have reported improved visual acuity (VA), but lacked detailed analysis of retinal structure and function in the treated area.

**Design:**

Phase 1/2 open-label dose-escalation trial to evaluate safety and potential efficacy (clinicaltrials.gov identifier, NCT01469832).

**Participants:**

Twelve participants with advanced Stargardt disease (STGD1), the most common cause of macular degeneration in children and young adults.

**Methods:**

Subretinal transplantation of up to 200 000 hESC-derived RPE cells with systemic immunosuppressive therapy for 13 weeks.

**Main Outcome Measures:**

The primary end points were the safety and tolerability of hESC-derived RPE cell administration. We also investigated evidence of the survival of transplanted cells and measured retinal structure and function using microperimetry and spectral-domain OCT.

**Results:**

Focal areas of subretinal hyperpigmentation developed in all participants in a dose-dependent manner in the recipient retina and persisted after withdrawal of systemic immunosuppression. We found no evidence of uncontrolled proliferation or inflammatory responses. Borderline improvements in best-corrected VA in 4 participants either were unsustained or were matched by a similar improvement in the untreated contralateral eye. Microperimetry demonstrated no evidence of benefit at 12 months in the 12 participants. In one instance at the highest dose, localized retinal thinning and reduced sensitivity in the area of hyperpigmentation suggested the potential for harm. Participant-reported quality of life using the 25-item National Eye Institute Visual Function Questionnaire indicated no significant change.

**Conclusions:**

Subretinal hyperpigmentation is consistent with the survival of viable transplanted hESC-derived RPE cells, but may reflect released pigment in their absence. The findings demonstrate the value of detailed analysis of spatial correlation of retinal structure and function in determining with appropriate sensitivity the impact of cell transplantation and suggest that intervention in early stage of disease should be approached with caution. Given the slow rate of progressive degeneration at this advanced stage of disease, any protection against further deterioration may be evident only after a more extended period of observation.

Embryonic stem cells are a potentially valuable source of donor cells for therapeutic repair and regeneration. Their pluripotency and innate capacity for self-renewal offer a virtually unlimited supply of highly specialized cells for therapeutic transplantation, but also present a potential risk of harm from uncontrolled proliferation and unintended differentiation. Their impact in experimental models has been investigated extensively, but evidence of their safety and potential efficacy in humans is limited. Embryonic stem cells have attracted attention for their potential value in a broad range of degenerative conditions, including those affecting the brain and eye. The eye has unique advantages as a target organ for cell transplantation. The retina is an extension of the brain that is readily accessible to surgical intervention under direct observation; its laminar structure lends itself to the targeted delivery of cellular therapies. The highly compartmentalized structure of the globe restricts potential dissemination locally and systemically. The intraocular environment is relatively protected against systemic immune responses that threaten allograft survival. Retinal microstructure can be observed in detail in the living eye noninvasively owing to the optical transparency of ocular media, and retinal function can be mapped topographically with exquisite sensitivity. The impact of intervention within a defined target region of the retina can be determined with confidence by comparison with untreated regions within the same eye and in the contralateral eye, which offer invaluable intraocular and intraindividual controls for the natural history of the condition and variability in performance.

Stargardt disease (STGD1) is the most common cause of macular degeneration in children and young adults. The condition originates in disease-causing sequence variants in the *ABCA4* gene^1^ and results in progressively severe impairment of sight. The *ABCA4* gene encodes a rim protein located on the intracellular disc membranes of light-sensitive photoreceptor cells that play an essential role in the retinoid cycle.^2^ Gene defects result in accelerated accumulation of a putative toxic metabolite, Di-retinoid-pyridinium-ethanolamine, within the underlying phagocytic retinal pigment epithelial (RPE) cells, leading to cell dysfunction and eventual cell death^3^ with progressive atrophy expanding from the central macula.^4^ Retinal pigment epithelial cells support the function and survival of overlying photoreceptor cells by multiple mechanisms, including recycling of visual pigment and phagocytosis of outer segments.^5^ Degeneration of RPE cells leads to secondary dysfunction and degeneration of overlying photoreceptor cells, and consequently progressively severe impairment of sight. Stargardt disease currently is untreatable, but replenishment of degenerating RPE cells with healthy cells offers the possibility of benefit by better supporting the function and survival of overlying photoreceptor cells, and consequently improving or protecting sight for a period limited by the consequences of the underlying photoreceptor disorder. A similar approach may benefit atrophic age-related macular degeneration, which shares key features with STGD1, including progressive atrophy of RPE and overlying photoreceptor cells. However, differences in their cause and progression are likely to influence the potential benefit of subretinal administration of human embryonic stem cell (hESC)-derived RPE cell suspensions. For example, age-related changes in Bruch’s (basement) membrane and chronic inflammation in age-related macular degeneration may influence the adhesion and survival of donor cells.

In experimental models of retinal degeneration, subretinal injection of hESC-derived RPE cell suspensions can protect photoreceptor cells and retinal function.^6^, ^7^ In human participants with STGD1, subretinal injection of up to 150 000 hESC-derived RPE cells resulted in no serious adverse events related to the transplanted cells.^8^, ^9^, ^10^ However, assessment of visual function has been limited. Herein we present the results of a dose-escalation trial of up to 200 000 hESC-derived RPE cells in 12 participants. To mitigate the risk of harm, we chose to investigate the safety of hESC-derived RPE transplantation in the poorer-seeing eye of individuals with advanced disease, acknowledging that any potential for benefit would be limited in this context by established degeneration of photoreceptor cells. Retinal degeneration in STGD1 typically advances progressively by expansion from the macula. We chose to administer cells to a target area, predefined for each study eye, extending from relatively well-preserved functional retina across a transitional zone of progressive degeneration to an area of atrophic nonfunctional retina. This afforded us the opportunity both to determine the safety of the transplanted cells in relatively healthy retina and to explore the potential benefit to function and survival of overlying photoreceptor cells in the degenerating and atrophic areas. By defining the target zone in the retina of each study eye before intervention, according to the distribution of disease severity and preferred locus of fixation, we were able to evaluate in detail the structure and function of this region before intervention using OCT, autofluorescence imaging, full-field perimetry, and microperimetry. For assessment of visual function, we established each participants’ test–retest variability for each test by means of multiple testing at baseline and controlled for the natural history of the condition, learning effects, and performance variability by comparison with the contralateral uninjected eye, on the basis that disease progression in an individual is symmetrical^11^ and that monocular performance of the contralateral uninjected eye is not influenced by the intervention. We considered that for each participant, changes in performance of both eyes symmetrically would indicate disease progression, learning effects, or both and that differences between the eyes may indicate a consequence of the intervention.

## Methods

### Trial Design

We performed a phase 1/2 open-label multicenter dose-escalation trial in 12 adult participants to evaluate the safety and tolerability of subretinal transplantation of hESC-derived RPE cells. The intervention was unmasked. The study received the approval of the Medicines and Health Products Regulatory Authority, the United Kingdom Gene Therapy Advisory Committee, and the Moorfields Eye Hospital Research Governance Committee. We provided potential candidates with detailed information, including an explanation of the aims of the trial and the possible consequences of participation. Participants provided their written informed consent before enrolment. We performed the study in accordance with the tenets of the Declaration of Helsinki and registered the trial at clinicaltrials.gov (identifier, NCT01469832).

### Trial Participants

We enrolled 12 participants with clinical features of STGD1, electroretinographic evidence of generalized rod and cone photoreceptor cell dysfunction, and molecular characterization of mutations in *ABCA4*. We excluded candidates in whom the visual acuity (VA) of their poorer-seeing eye was 20/400 or better, those with any history of malignant neoplasia or significant ocular pathologic features other than STGD1, any contraindication to systemic immunosuppression or surgery under general anaesthesia, and women who were pregnant or lactating. We enrolled participants sequentially into escalating dose groups of 50 000, 100 000, 150 000, and 200 000 hESC-derived RPE cells. Each dose group included 3 participants. For each participant, we selected the poorer-seeing eye (according to dominance and VA) as the study eye for intervention; the better-seeing contralateral eye served as an untreated control for intraindividual natural history and performance variability.

### Intervention

Retinal pigment epithelial cells were generated from the hESC line MA09 in accordance with good manufacturing practice. The hESC bank was thawed and expanded on mitomycin C–treated mouse embryonic fibroblasts for 3 passages. Human embryonic stem cells were dissociated and seeded to allow embryoid body formation. Pigmented RPE patches then were isolated with collagenase from cellular outgrowths. After purification and trypsinization, the RPE cells were expanded and cryopreserved at passage 2 for clinical application. We characterized RPE cells in process and after freezing and formulation, including karyotyping, pathogen and phagocytosis assay testing, and differentiation and purity evaluation by morphologic assessment, quantitative polymerase chain reaction, and quantitative immunohistochemistry for RPE and hESC markers. On the day of transplantation, cells were thawed from cryopreserved hESC-derived RPE cell banks, formulated at the appropriate concentration, stained to exclude bacterial contamination, and delivered to the operating room for administration.^8^

The surgical procedure was conducted under general anaesthesia. We performed 3-port pars plana vitrectomy, with separation of the posterior hyaloid from the posterior pole toward the equator. Administration of hESC-derived RPE cells was preceded by subretinal injection of up to 0.2 ml Hartmann’s solution using a 41-gauge cannula to establish the target tissue plane and to minimize unintended administration of cells into the vitreous or choroid. The hESC-derived RPE cell suspension, reconstituted to the appropriate concentration in 150 μl, was injected using a 38-gauge subretinal cannula (MedOne PolyTip Cannula 23/38; MedOne Surgical, Sarasota, FL) into the preformed bleb of Hartmann’s solution. After subretinal injection, we performed a washout of the vitreous cavity by continuous infusion and aspiration of fluid to minimize the presence of donor cells remaining in this compartment. Participants were advised to maintain a supine position for 6 hours after surgery.

Human embryonic stem cell-derived RPE cells were delivered into a preselected transition region of the retina, defined for each participant as a region extending from an area of relatively preserved structural integrity and function to an area of atrophic nonfunctional retina. The rationale was to investigate the safety of hESC-derived RPE cell administration in areas of relatively well-preserved retina and the potential benefit in areas of retinal dysfunction and atrophy. The target region was defined before surgery for the study eye of each participant with regard to the distribution of the disease severity and preferred locus of fixation using spectral-domain (SD) OCT, fundus autofluorescence (FAF) imaging, and microperimetry. We defined target regions close to the preferred locus of fixation to optimize the quality of structural and functional assessment.

To manage the risk of immune rejection of the transplanted hESC-derived RPE cells, participants were prescribed oral immunosuppression in the perioperative period. Low-dose tacrolimus (0.1 mg/kg) was divided into 2 daily doses from 1 week before transplantation, titrated to achieve trough serum levels of 3 to 7 ng/ml, and maintained until week 6 after transplantation, when it was discontinued. Mycophenolate was commenced on the day of surgery (day 0) in an escalating dose regimen: 0.25 g twice daily (day 0 to day 1); 0.5 g twice daily (day 2 to day 3), and 1 g twice daily from day 4 until 12 weeks after transplantation, when it was discontinued. Participants also were prescribed a standard postvitrectomy regimen of topical antibiotics (chloramphenicol 0.5% 4 times daily for 7 days) and topical nonsteroidal anti-inflammatory drugs (ketorolac 0.5% 4 times daily for 4 weeks).

### Outcome Measures

The primary end points were the safety and tolerability of hESC-derived RPE cells as defined by the absence of any grade 2 or more (National Cancer Institute grading system) adverse event relating to the transplanted cells, any evidence that the cells were contaminated with an infectious agent, any evidence that the cells showed potential of tumorigenicity, or a combination thereof. Secondary end points included safety of the surgical procedure, dose selection for future studies, evidence of donor cell survival and engraftment in the target location, and retinal function as measured by electroretinography. Exploratory end points included VA, color discrimination, retinal sensitivity by perimetry, and quality of life (assessed with the 25-item National Eye Institute Visual Function Questionnaire).

We evaluated participants at baseline and after hESC-derived RPE cell transplantation at intervals for 12 months. We performed examination by slit-lamp biomicroscopy and indirect binocular ophthalmoscopy, color fundus photography, FAF imaging, fundus fluorescein angiography, and SD OCT. We measured best-corrected VA (BCVA) and color discrimination (Universal Colour Discrimination Test) and performed microperimetry (Nidek MP-1, NIDEK Inc, Fremont, CA) full-field static perimetry (Octopus 900, Haag-Streit, Switzerland), Goldmann kinetic perimetry, and electroretinography (including full-field, pattern, and multifocal electroretinography to international standards using gold-foil electrodes). We used standardized protocols and a fixed sequence of test patterns. We assessed participants’ systemic health by physical examination results, electrocardiography results, cancer screening results, and hematologic and serologic results. After completion of the initial 12-month evaluation period, participants were followed up for long-term safety assessments, the anticipated duration of which is lifelong.

### Statistical Analysis

The small sample size was not designed for statistical analysis. The end points for efficacy were solely descriptive and were defined as any improvement in visual function beyond the test–retest variability within the full cohort for each assessment, determined by means of 1-way analysis of variance with the use of multiple measurements at baseline.^12^

### Role of the Funding Source

The funder of the study participated in the study design, data analysis and interpretation, and writing of the report. The corresponding author had full access to all the data in the study and had final responsibility for the decision to submit for publication.

## Results

### Administration of Human Embryonic Stem Cell-Derived Retinal Pigment Epithelial Cell Suspension

**Figure 1:**
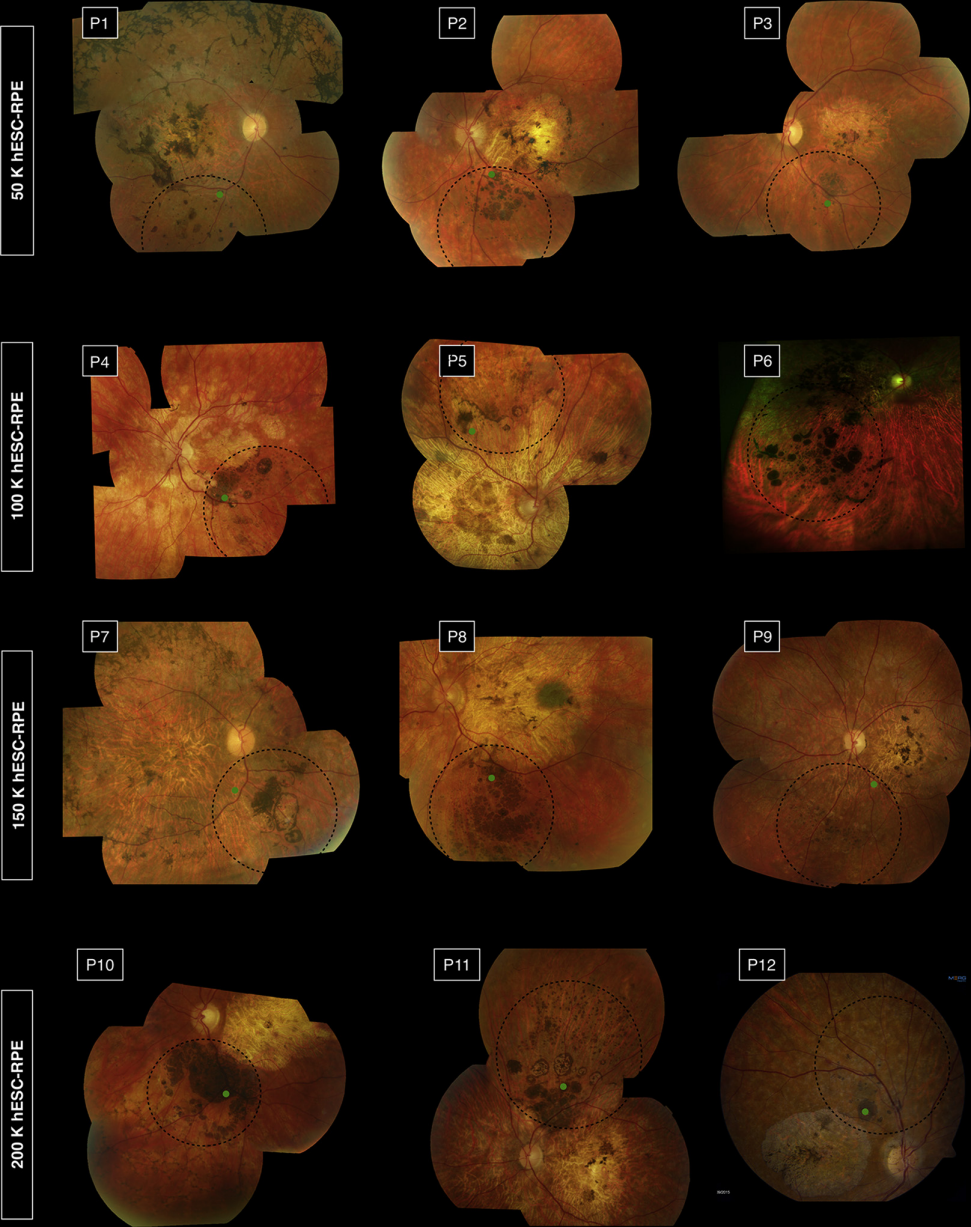
Fundus photographs of recipient eyes at 12 months after transplantation. In each image, the retinotomy site is indicated by the green dot, and the area of subretinal administration is outlined by the dotted black line. P = patient.

We administered hESC-derived RPE cells to 12 participants (age range, 34–53 years) with molecularly proven advanced STGD1 (Table 1). The hESC-derived RPE cells were reconstituted and administered subretinally by 2 of the authors—patients 1 through 11 by J.W.B.B. and patient 12 by D.S.—to the predefined target regions, which were inferior to the inferotemporal vascular arcade in 10 eyes and superior to the superotemporal arcade in 2 eyes (patients 5 and 11; Fig 1). We performed intraoperative prophylactic retinopexy for pre-existing pigmented retinal breaks in 2 participants (patients 3 and 5) and for atrophic retinal holes in 2 participants (patients 10 and 11). In 1 participant (patient 12), cryoretinopexy and injection of sulphahexafluouride gas were performed for 2 small superonasal retinal tears and a small inferotemporal retinal dialysis with localized avulsion of pars plana epithelium. In all eyes, the bleb of subretinal fluid resorbed clinically within 24 hours, with no persistent or unplanned retinal detachment. Small subretinal hemorrhages at the injection sites were evident in 2 participants (patients 1 and 12). Mild postoperative vitreous cavity hemorrhage in 1 participant (patient 5), arising from a pars plana entry site, cleared spontaneously within 4 weeks. A mild asymptomatic posterior subcapsular lens opacity was evident in 1 participant (patient 6) at month 9. Adverse events related to immunosuppression in 5 participants (patients 5, 6, 7, 10, and 12) included lethargy, headache, nausea, and herpes simplex virus 1 (HSV1) reactivation. All participants completed 12 weeks of immunosuppression after transplantation.

**Table 1:** Participants and Dosing

Participant number	Age (yr)	hESC-RPE dose	Sex	Study eye	Baseline Visual Acuity ETDRS LogMAR		Genotype Pathogenic variants on ABCA4 gene
					Study eye	Control eye	
P1	34	50 000	M	OD	1.29	1.23	c.768G>T (p.?) c.5461-10T>C (p.?)
P2	44		M	OS	1.36	1.39	c.4918C>T (p.Arg1640Trp) c.6079C>T (p.Leu202Phe)
P3	46		M	OS	1.33	1.33	c.2588G>C (p.Gly863Ala) c.5461-10T (p.?) c.2828G>A (p.Arg943Gln) c.5603A>T (p.Asn1868Ile)
P4	41	100 000	M	OS	1.39	1.35	c.4139C>T (p.Pro1380Leu) c.5461-10C>T (p.?)
P5	51		M	OD	1.37	1.39	c.454C>T (p.Arg152*) c.1715G>A (p.Arg572Gln) c.2588G>C (p.Gly863Ala) c.6148G>C (p.Val2050Leu)
P6	44		F	OD	1.84	1.84	c.161G>A (p.Cys54Try) c.4462T>C (p.Cys1488Arg)
P7	40	150 000	M	OD	1.84	1.36	c.666_678del (p.Lys223fs) c.5461-10T>C (p?)
P8	53		M	OS	1.27	1.23	c.2588G>C (p.Gly863Ala) c.2828G>A (p.Arg943Gln) c.5603A>T (p.Asn1868Ile)
P9	40		M	OS	1.39	1.33	c.634C>T (p.Arg212Cys) c.4319T>C (p.Phe1440Ser)
P10	50	200 000	M	OS	1.25	1.19	c.1906C>T (p.Gln636*) c.2588G>C (p.Gly863Ala)
P11	45		M	OS	1.39	1.39	c.5461-10T>C c.5929G>A (p.Gly1977Ser)
P12	45		M	OS	1.09	1.10	c.2588G>C (p.Gly863Ala) c.4469G>A (p.Cys1490Tyr)

We identified no evidence of acute immune rejection such as vitreitis, retinitis, retinal exudates, retinal edema, or vascular hyperpermeability. In no instance was intraretinal fluid evident on OCT, and no change of clinical concern was apparent on fundus fluorescein angiography. We found no evidence of adverse proliferation of transplanted hESC-derived RPE cells either intraocularly or on systemic screening by clinical examination (including testicular and prostate examination in men and breast and pelvic examination in women) or investigation (including fecal occult blood test, chest radiography, thyroid function analysis, prostate-specific antigen analysis, and cervical smear analysis, as appropriate).

### Effect of Human Embryonic Stem Cell-Derived Retinal Pigment Epithelial Cell Transplantation on Retinal Structure

Within 4 weeks of administration of hESC-derived RPE cells, subretinal hyperpigmentation developed within the area of injection of the cell suspension in all 12 participants (Fig 1). The mean area of subretinal hyperpigmentation increased progressively, at a rate that seemed to be dose dependent during the first 3 months, and subsequently slowed (Fig S1, available at www.aaojournal.org). A subsequent modest reduction in area of subretinal hyperpigmentation was evident in 2 participants (patients 4 and 9). The mean area of subretinal pigmentation at 12 months was correlated directly with the dose of hESC-derived RPE cells administered (*R*^2^ = 0.981; Fig S2, available at www.aaojournal.org).

**Figure 2:**
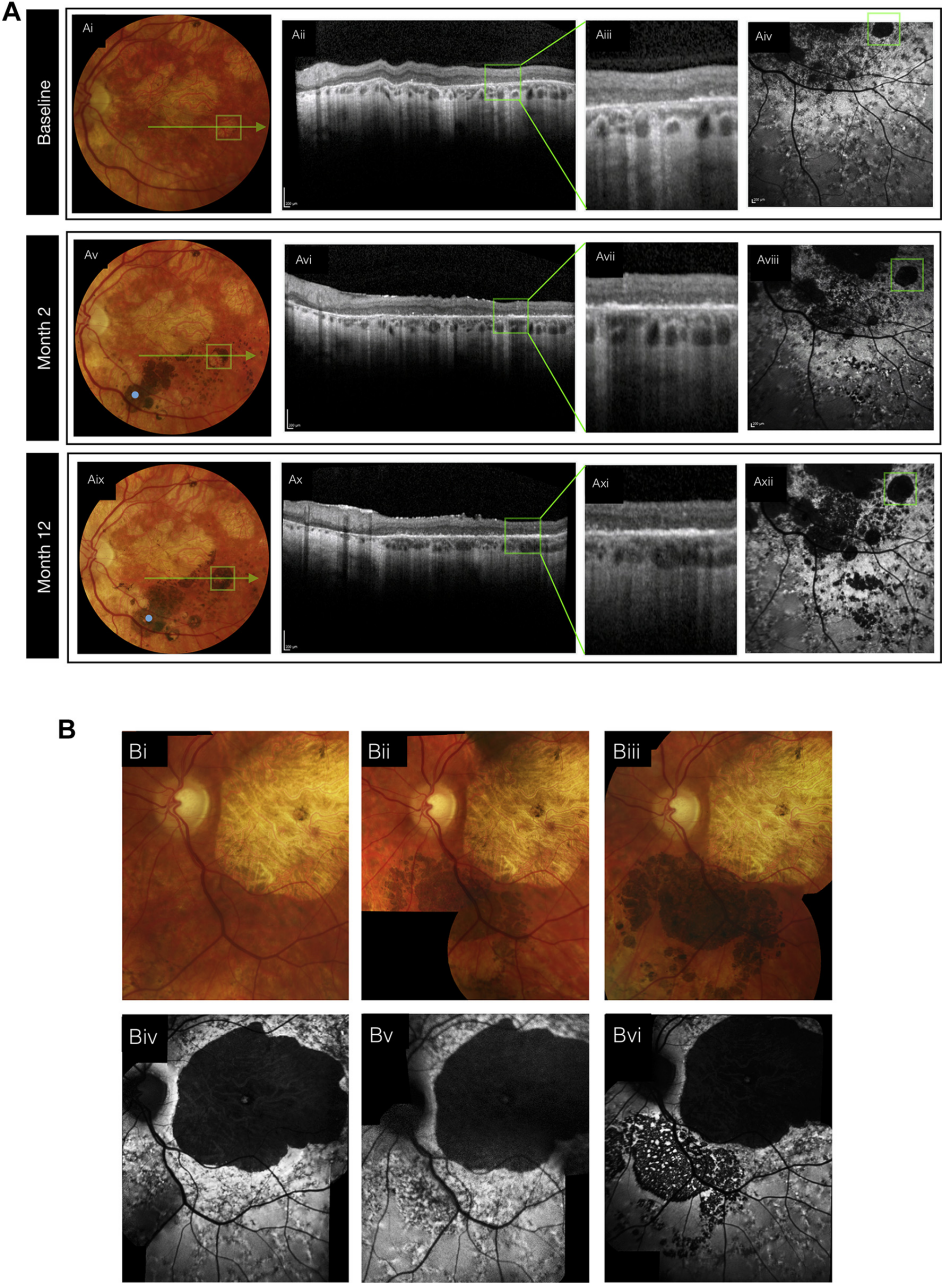
Fundus photographs, OCT images, and fundus autofluorescence images in (**A**) patient 4 and (**B**) patient 10. **A**, In patient 4, fundus images demonstrating the time-course of hyperpigmentation (**Ai**, **Aiv**, and **Avii**) are presented with corresponding OCT line scans, at lower and higher magnification. A progressive increase in optical signal evident in the outer retina consistent with the continued presence of transplanted cells. **B**, In patient 10, images demonstrating the time-course of hyperpigmentation (**Bi**, **Bii**, and **Biii**) are presented with the associated fundus autofluorescence images demonstrating reduced signal consistent with masking of endogenous autofluorescence.

Areas of subretinal hyperpigmentation corresponded on SD OCT to a hyperreflective signal located between the photoreceptor cell layer and the underlying Bruch’s membrane that persisted for 12 months in all eyes. Focal changes in FAF, which may be an indirect indication of RPE cell function, were evident within the region of cell administration. A relative predilection for the development of hyperpigmentation at sites of retinal atrophy within the injected area was evident. In 1 patient (patient 4), a circular region of atrophic retina, which on SD OCT appeared to be devoid of RPE, developed progressively confluent hyperpigmentation with increasing subretinal hyperreflectivity on SD OCT and masking of choroidal hyperreflectivity (Fig 2A). However, recovery of FAF in this area was undetectable at 12 months (Fig 2Axii). Hyperpigmentation in areas overlying surviving host RPE typically was associated with reduced FAF, consistent with masking of the autofluorescence of underlying host RPE (Fig 2B). We investigated the relationship between hyperpigmentation and retinal thickness using SD OCT in the 3 participants (patients 8, 9, and 10) whose fixation stability enabled reliable serial analysis; in 2 participants (patients 8 and 9), we identified no clear relationship; in 1 participant (patient 10), who was administered the highest dose, retinal thinning was evident in the hyperpigmented areas at 12 months (Fig S3, available at www.aaojournal.org).

Focal preretinal pigmentation (on the inner surface of the retina) developed in 2 participants (Fig 1). In 1 participant (patient 1), preretinal pigmentation was associated with limited adherence to advice to maintain a supine position after surgery; in this instance, the area of preretinal pigmentation increased progressively during the 12-month period, but subsequently stabilized (data not shown). One participant (patient 12) demonstrated a nonpigmented and noncontractile epiretinal membrane over the injection site at month 6; this subsequently stabilized in extent with no measurable effect on visual function. Focal pigmented deposits were evident in the vitreous cavity in 2 participants (patients 6 and 7), both of whom reported visual floaters.

### Effect of Human Embryonic Stem Cell-Derived Retinal Pigment Epithelial Cell Transplantation on Retinal Function

We measured no clinically significant decline or improvement in retinal function by electroretinography in any of the participants during the 12-month period. Participant-reported quality of life using the 25-item National Eye Institute Visual Function Questionnaire indicated no significant change. A transient reduction in BCVA of operated eyes was evident in 7 participants (patients 2, 3, 4, 5, 8, 11, and 12) after surgery and resolved within 9±6 days (range, 3–21 days; Fig S4, available at www.aaojournal.org). Borderline improvements in BCVA were evident in 4 participants, although these were either unsustained (patients 1, 7, and 10) or matched by a similar improvement in the contralateral eye (patient 12). The mean BCVA of the 150 000-cell dose improved to the limit of baseline variability; for the other dose groups, the mean BCVA remained within the limits of test–retest variability during the 12-month period. The participants’ preferred loci of fixation were unaltered (data not shown).

We investigated retinal sensitivity in detail using mesopic microperimetry (Nidek MP-1). We measured retinal sensitivity at loci in the macular area (central 20°) in both the study eye (including a variable portion of the transplanted area) and contralateral eye (in all except patient 12) and calculated the change in sensitivity from baseline (Fig S5, available at www.aaojournal.org). A borderline improvement in retinal sensitivity of the study eye in 1 participant (patient 7) was evident at 3 months only. The mean retinal sensitivity in all 4 dose groups remained within the limits of the test–retest variability.

**Figure 3:**
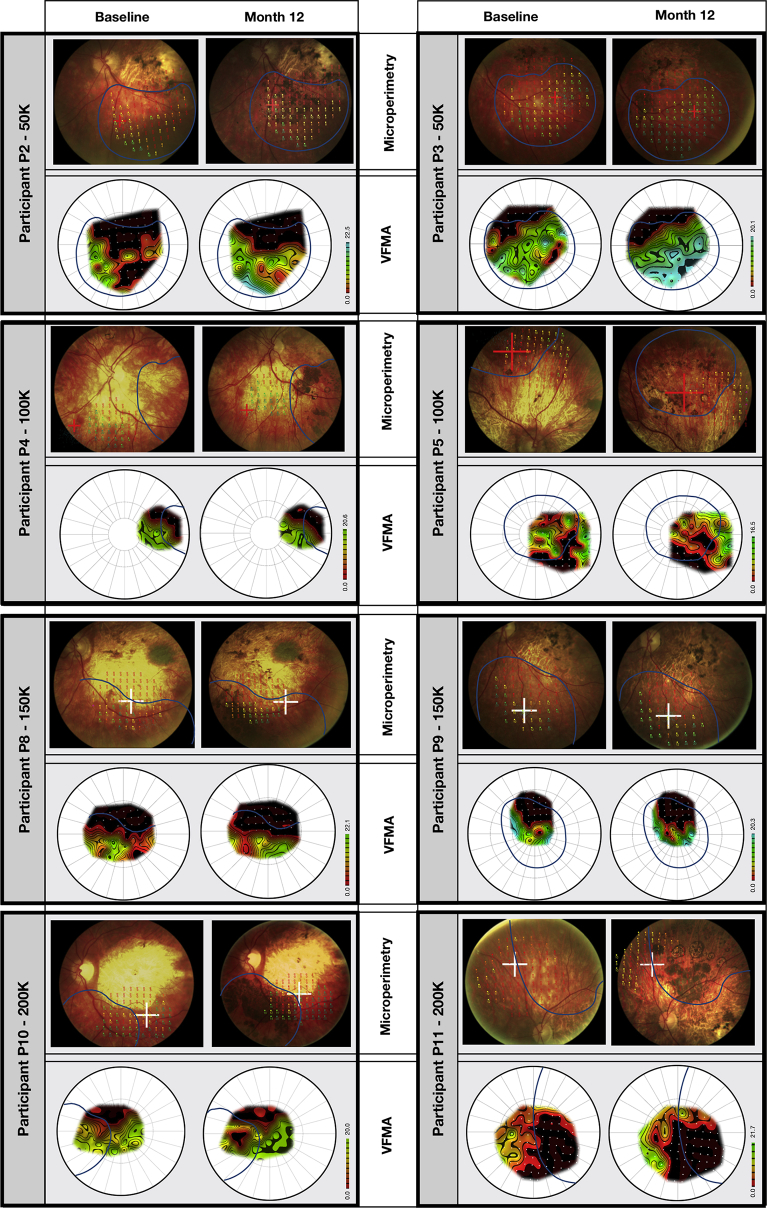
Microperimetry images showing topography of retinal sensitivity. The retinal sensitivities at test loci in each study eye, measured by microperimetry (Nidek MP-1) at baseline and 12 months, are superimposed on the respective fundus image. Each is presented with a corresponding topographic contour map to illustrate the hill of vision of retinal sensitivity, constructed by interpolation of sensitivities at the test loci using Visual Field Modelling and Analysis (VFMA) software. The blue line outlines the area of recipient retina administered human embryonic stem cell-derived retinal pigment epithelial cell suspension. P = patient; K = thousand.

**Figure 4:**
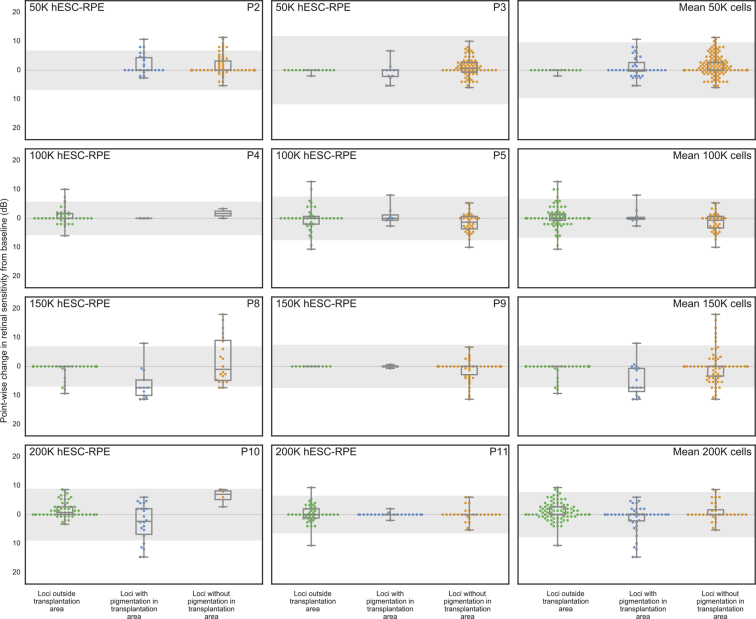
Boxplots showing the change in retinal sensitivity from the baseline (mean of 3 baseline tests) as measured by microperimetry (Nidek MP-1) for each test locus at month 12. Loci were stratified into 3 groups: outside the transplantation area (solid green dots), within the transplantation and associated with subretinal hyperpigmentation (solid blue dots), or within the transplantation area and without subretinal hyperpigmentation (solid orange dots). Using a selection tool on the Visual Field Modelling and Analysis (VFMA) software, volumetric sensitivity within regions of retinal pigmentation were extracted from regions of pigmentation and nonpigmentation. The boxplots show the median value, quartiles, and range. hESC = human embryonic stem cell; K = thousand; P = patient; RPE = retinal pigment epithelial.

For the study eyes of 8 participants (all except patients 1, 6, 7, and 12, for whom the assessment was unavailable or unreliable) we measured in greater detail, using a stimulus grid of high density, the sensitivity of the area of retina specifically targeted with the hESC-derived RPE cell suspension. The total retinal sensitivities of each eye and the mean sensitivities of each dose group remained within the limits of test–retest variability during the 12-month period (Fig S6, available at www.aaojournal.org). To quantify the spatial correlation of retinal sensitivity with hyperpigmentation, we measured the change in retinal sensitivity at 12 months at hyperpigmented and nonhyperpigmented loci (Fig 3). The retinal sensitivity at most loci remained within the limits of variability, whether within or outside the transplanted area, and regardless of the presence of hyperpigmentation (Fig 4); in 2 participants administered higher doses (patients 8 and 10), retinal sensitivity at hyperpigmented loci tended to be reduced. To demonstrate highly localized changes in retinal sensitivity, we derived corresponding topographic contour maps using Visual Field Modelling and Analysis software^13^ (Fig 3). Localized areas of improved retinal sensitivity (for example, in patients 2, 3, 8, 9, and 11) were not hyperpigmented, whereas retinal sensitivity in areas of hyperpigmentation appeared unchanged or reduced (for example, in patients 8 and 10).

We calculated the mean full-field retinal sensitivity in both the study eyes and control eyes of each dose cohort (in all participants except patients 6 and 12) by full-field static perimetry (Octopus 900). Deterioration in full-field sensitivity was evident in both the study and control eyes of 2 participants (patients 2 and 4), and an associated reduction in both the study and control eyes of the 100 000-cell dose cohort to the lower limit of test–retest variability (Fig S7, available at www.aaojournal.org). The individual and mean sensitivities otherwise were maintained within the limits of variability for the 12-month period.

Because participants in the early stages of the trial described subjective improvements after intervention in their sensitivity of their study eye to color, we measured color vision in a subset of 4 participants (patients 8–11) using the Universal Colour Discrimination Test. A single assessment at baseline demonstrated poor color discrimination in both eyes. Serial assessments after intervention indicated no improvement in color vision that was specific to the study eye (Fig S7).

## Discussion

Stargardt disease causes progressive and irreversible macular degeneration and severe impairment of sight.^14^ Repair of the compromised or degenerate RPE by transplantation of healthy RPE cells offers the possibility of protecting or improving sight by supporting the function or survival of overlying photoreceptor cells. The results of the trial satisfied its primary end point for safety, with no evidence of inflammatory responses or uncontrolled intraocular or systemic proliferation, after subretinal administration of up to 200 000 hESC-derived RPE cells. There were no serious adverse events related to the surgical procedure; intraoperative adverse events resulted in no harm to visual function beyond the immediate postoperative period. A brief decline in BCVA in the operated eye after administration of hESC-derived RPE cells was a predictable temporary consequence of intraocular surgery. The development of pigmented epiretinal membranes after injection of hESC-derived RPE cells has been reported previously^9^ and is consistent with reflux of cell suspension from the subretinal space. Although we measured no associated adverse impact on visual function at 12 months, the longer-term significance has yet to be determined. Adverse events related to the immunosuppressive medications occurred during the period of their administration, but had no impact subsequently.

The development of subretinal hyperpigmentation within the injected area in all 12 participants was dose dependent and associated in many instances with a hyperreflective signal on OCT, suggesting survival of injected donor hESC-derived RPE cells. The high density of pigmentation in these areas may reflect a greater melanin content, higher density, hypertrophy,^15^ or multilayering of surviving donor hESC-derived RPE cells. The presence of pigmented subretinal material is not definitive evidence of donor cell survival and alternatively may reflect persistence of melanin pigment after their death,^16^, ^17^ a consequence of transplantation that equally could be dose dependent. We found no definitive evidence of local accumulation of lipofuscin on FAF imaging to demonstrate physiologic activity, and hence viability, of the cells, although FAF may take longer than 12 months to develop. Preferential engraftment of RPE cell transplants to areas devoid of host RPE is plausible given the anchorage-dependent properties of RPE cells^18^, ^19^ and similarly has been described in experimental in vivo studies.^20^ The progressive local extension of hyperpigmentation during the first 3 months and the predilection for areas of atrophy in the recipient retina are consistent with local migration of surviving donor cells, but may reflect subretinal migration of released pigment.

To mitigate the risk of immune rejection of injected allogenic hESC-derived RPE cells, we administered systemic immunosuppressive medications to all participants for 13 weeks. We identified no overt clinical signs to suggest acute or delayed rejection. Specifically, we noted no vitreitis, retinal exudation or retinitis, vascular leakage, choroiditis, or cystoid macular edema, although features of rejection may not always be readily evident.^21^ We identified no change on clinical examination or imaging after withdrawal of immunosuppression, 12 weeks after the injection procedure. A reduction in the pigmentation density at the site of transplantation, evident in 2 participants (at month 6 in patient 9 and at month 12 in patient 4) may reflect rejection of pigmented donor hESC-derived RPE cells, although no associated change in retinal function was apparent. The risk of immune rejection may be reduced by use of autologous RPE cells derived from induced pluripotent stem cells, although such cells nonetheless may be subject to rejection, and the process of cell derivation specific to an individual presents considerable logistical challenges.^22^

Reports of vision loss after intravitreal injection of autologous adipose tissue-derived stem cells highlight the potential for harm resulting from transplantation of cell populations that are poorly characterized and controlled.^23^ In our study, intraocular administration of carefully obtained hESC-derived RPE cells resulted in the development of pigmented foci in the vitreous cavity in 2 participants and on the surface of the inner retina in 2 participants, suggesting reflux of donor hESC-derived RPE cells from the subretinal compartment into the vitreous cavity. Despite the presence of preretinal or intravitreal pigmentation, no associated adverse effect was evident.

The aim of intervention is to protect or improve aspects of sight. In the current trial, all participants showed advanced disease before intervention, with established retinal degeneration and severe impairment of visual function. We measured no consistent progression of disease during the 12-month trial; deterioration in the visual fields of both eyes of 2 participants (patients 2 and 4) was apparent on full-field perimetry, but was not evident on microperimetry. Given the slow rate of progressive degeneration at this advanced stage of disease, any protection against further deterioration may be evident only after a more extended period of observation.

We also sought to determine whether the function of photoreceptor cells that are surviving but compromised may improve after the provision of healthy supporting RPE cells. In a previous trial of up to 150 000 hESC-derived RPE cells in STGD1, variable improvements in VA were measured in 3 of 8 eyes without secondary cataract, although the mean difference in VA between the recipient and contralateral eyes was not statistically significant.^9^ In a subsequent trial in 2 participants with STGD1, improvements in VA were reported in both the recipient and contralateral eyes.^10^ In the present trial, we administered up to 200 000 hESC-derived RPE cells and measured retinal structure and function in detail using microperimetry and SD OCT. Our findings demonstrate the value of detailed analysis of spatial correlation of retinal structure and function in determining with appropriate sensitivity the impact of cell transplantation. We found no significant benefit to retinal function at 12 months in the 12 participants. Although RPE cell transplantation theoretically may improve the function of overlying photoreceptor cells, the potential for improvement is limited in this particular population of participants by the severity of established retinal degeneration.

The evidence of safety broadly supports the rationale for further studies to explore the impact of intervention at an earlier stage of degeneration when surviving photoreceptors cells may stand to benefit with improved function and survival. However, instances of focally reduced sensitivity and thinning in hyperpigmented retina at higher doses of hESC-derived RPE cells suggest the potential for harm and indicate that intervention at earlier stages of degeneration should be approached with caution.
