# Supplementary material for: Transplantation of Human Embryonic Stem Cell-Derived Retinal Pigment Epithelial Cells in Macular Degeneration
Source: Ophthalmology. 2018 Nov;125(11):1765–75. doi: 10.1016/j.ophtha.2018.04.037 (PMC6195794; doi:10.1016/j.ophtha.2018.04.037)
Supplement: Figure S1 [file mmc1.pdf]

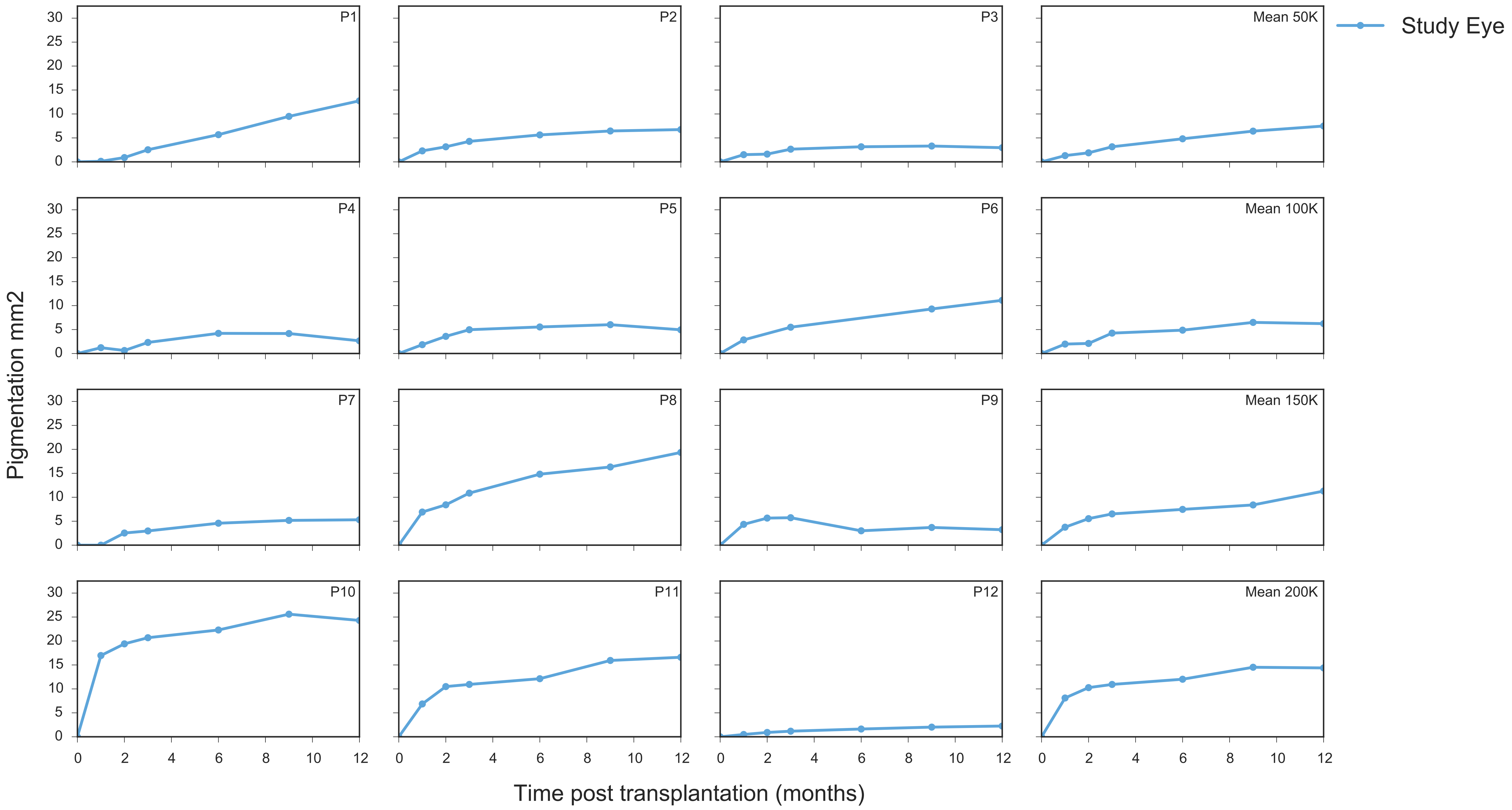

Supplementary Figure 1: Time-course of pigmentation

The total area of sub-retinal pigmentation in the study eye of each participant was quantified using ImageJ software.
