# Supplementary material for: Transplantation of Human Embryonic Stem Cell-Derived Retinal Pigment Epithelial Cells in Macular Degeneration
Source: Ophthalmology. 2018 Nov;125(11):1765–75. doi: 10.1016/j.ophtha.2018.04.037 (PMC6195794; doi:10.1016/j.ophtha.2018.04.037)
Supplement: Figure S2 [file mmc2.pdf]

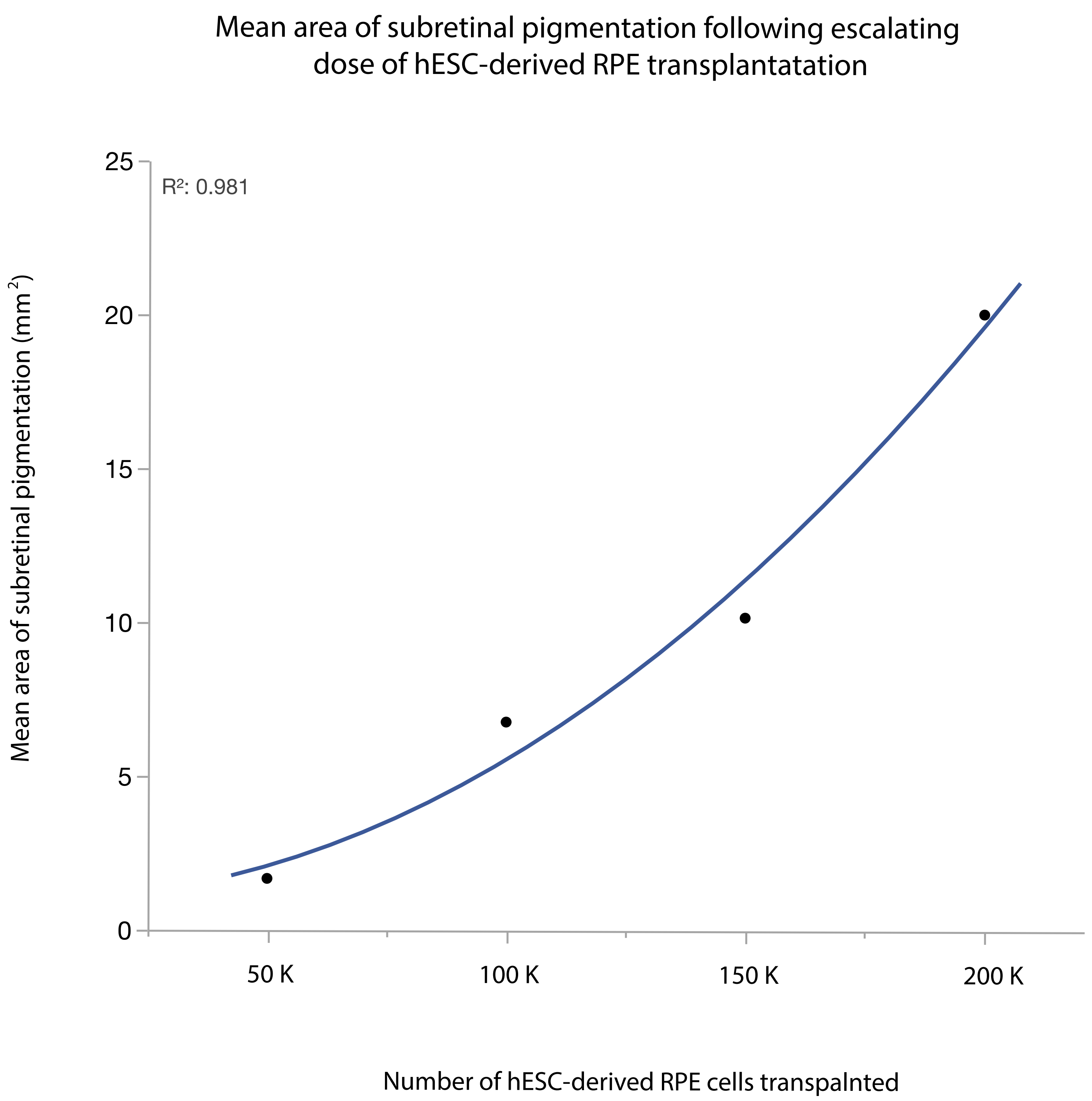

Supplementary Figure 2: Pigmentation-dose response

The mean area of subretinal pigmentation correlated directly with the number of hESC-RPE cells transplanted ($R^2 = 0.981$)
