# Supplementary material for: Transplantation of Human Embryonic Stem Cell-Derived Retinal Pigment Epithelial Cells in Macular Degeneration
Source: Ophthalmology. 2018 Nov;125(11):1765–75. doi: 10.1016/j.ophtha.2018.04.037 (PMC6195794; doi:10.1016/j.ophtha.2018.04.037)
Supplement: Figure S3 [file mmc3.pdf]

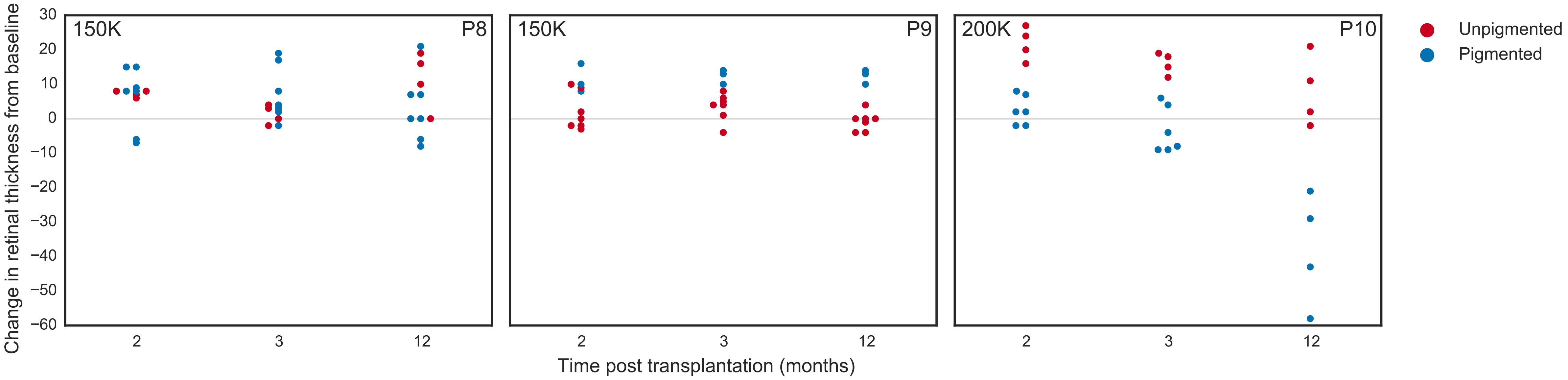

Supplementary Figure 3: Retinal thickness

We performed Spectral-Domain OCT (SD-OCT, Spectralis, Heidelberg Engineering, Germany) volume-scans at the horizontal meridian in all participants across the area of cell transplantation. The volume acquisition protocol involved 241-B scans covering an area of 30° by 25°. Where possible, follow-up OCT images were acquired by means of the automated 'Follow-up' function using the automatic real time tracking function. Retinal thickness was determined using the automated retinal thickness measurements between the inner limiting membrane (ILM) and the inner border of the line of back-reflection indicating the RPE; this was checked for segmentation errors. Multiple measurement of retinal thickness was taken at defined loci (spaced at 1000μm) within the region of subretinal transplantation. Each locus was than classified as 'non-hyperpigmented' or 'hyperpigmented' depending on the presence of hyperpigmentation at final month 12 imaging. Of the 12 participants, only 3 (P8, 9 and 10) maintained adequate stability of fixation to enable reliable serial follow using the 'Follow-up' acquisition function.
