# Supplementary material for: Transplantation of Human Embryonic Stem Cell-Derived Retinal Pigment Epithelial Cells in Macular Degeneration
Source: Ophthalmology. 2018 Nov;125(11):1765–75. doi: 10.1016/j.ophtha.2018.04.037 (PMC6195794; doi:10.1016/j.ophtha.2018.04.037)
Supplement: Figure S4 [file mmc4.pdf]

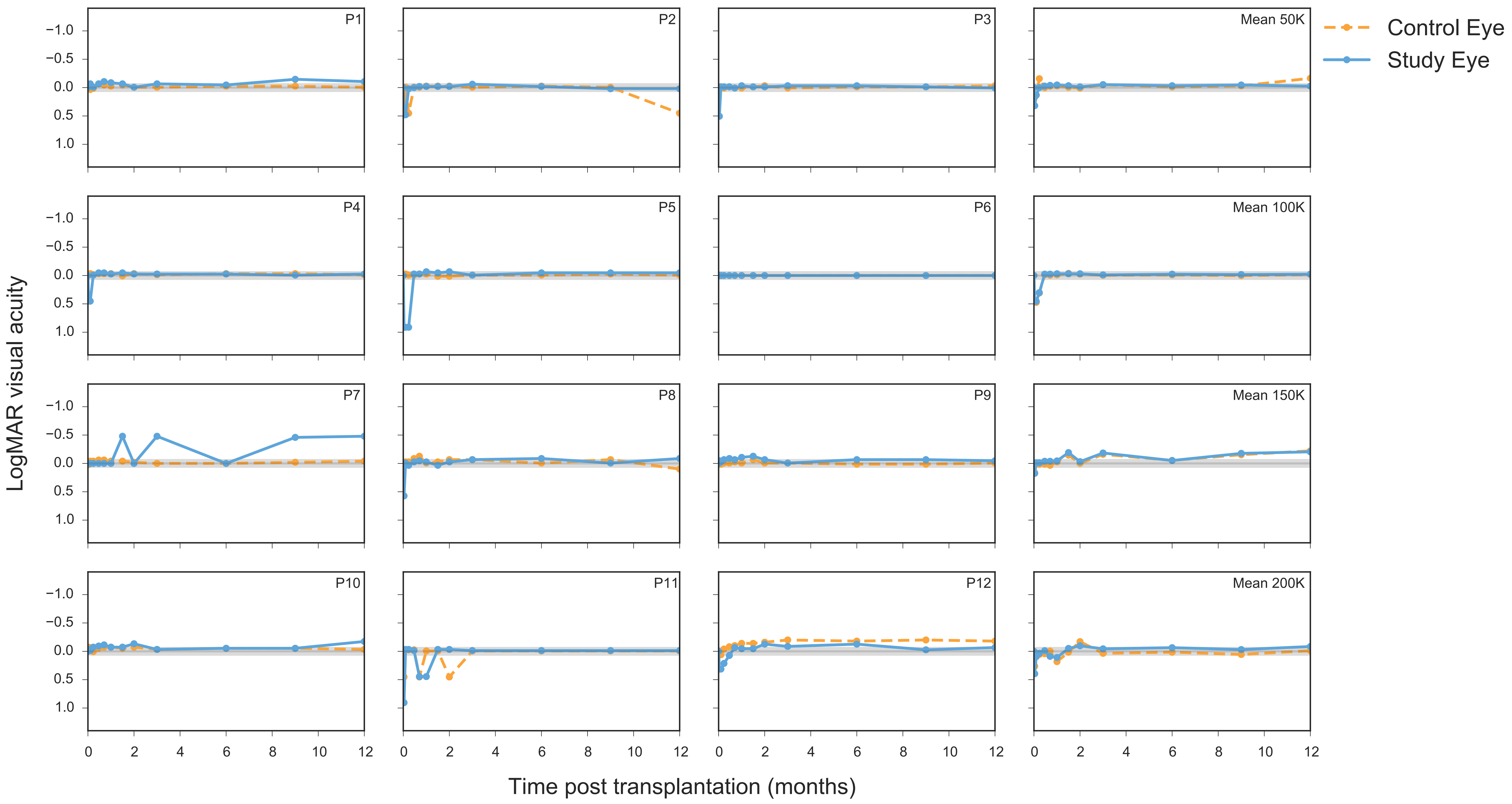

Supplementary Figure 4: Visual acuity

For each of the 12 participants, a continuous blue line indicates the visual acuity of the study eye, and a dotted orange line indicates that of the contralateral control eye. Baseline visual acuity is presented as the mean of 3 separate measurements. The grey areas indicate test-retest variability, determined using multiple baseline measurements with the one-way ANOVA method.[1]

References

1. Bland JM, Altman DG. Measurement error. BMJ 1996;312:1654.
