# Supplementary material for: Transplantation of Human Embryonic Stem Cell-Derived Retinal Pigment Epithelial Cells in Macular Degeneration
Source: Ophthalmology. 2018 Nov;125(11):1765–75. doi: 10.1016/j.ophtha.2018.04.037 (PMC6195794; doi:10.1016/j.ophtha.2018.04.037)
Supplement: Figure S5 [file mmc5.pdf]

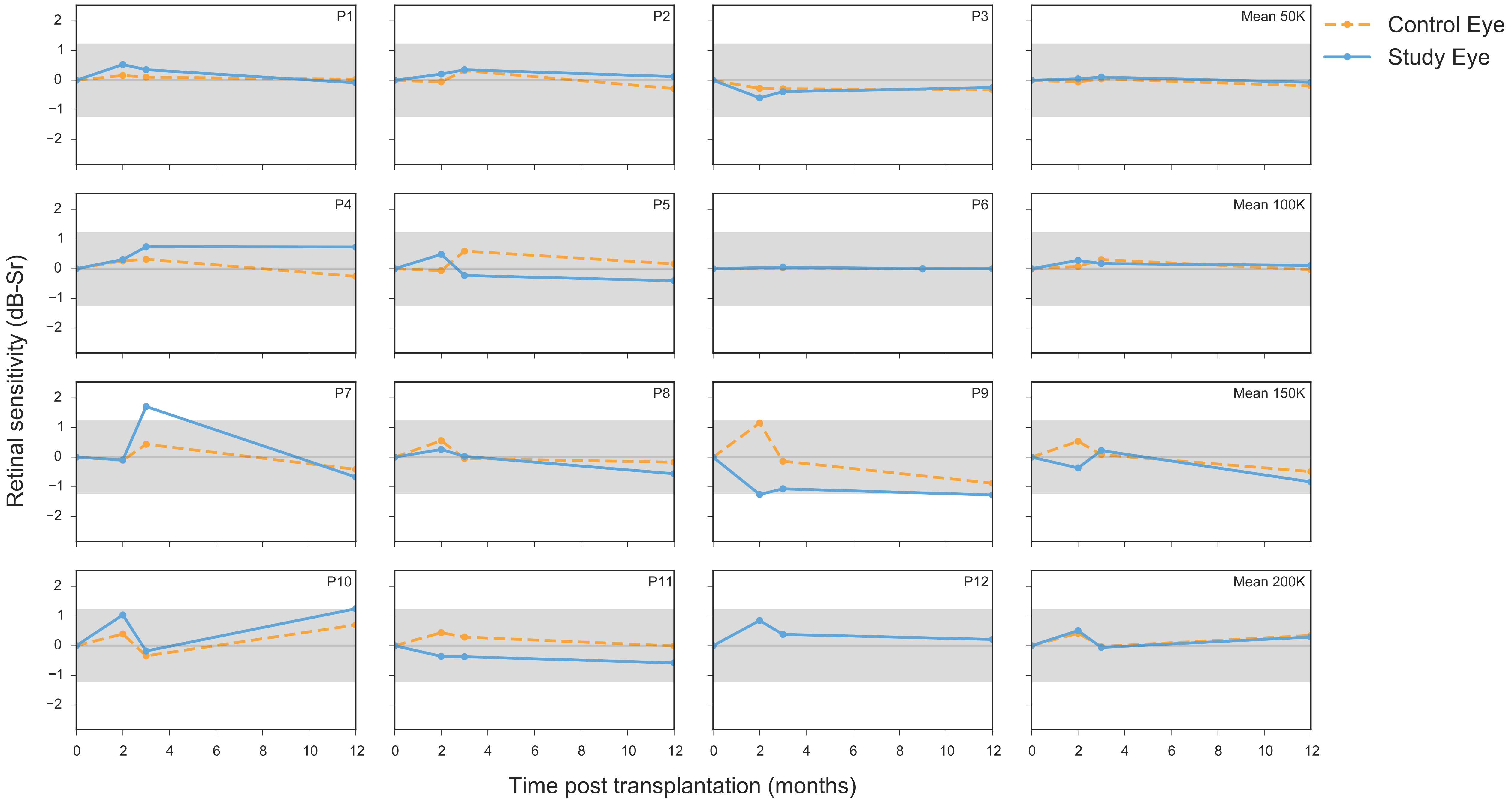

Supplementary Figure 5: Retinal sensitivity by wide-field microperimetry

Volumetric retinal sensitivity in dB-sr plotted over 12 months for each participant. Sensitivities from each test locus of a central macular grid (Humphrey 10-2 grid) were interpolated to produce a 3-dimensional topographic map and quantified volumetrically using Visual Field Modelling and Analysis (VFMA) software developed by one of the authors.[2] Data from study eyes are plotted with a filled blue symbol connected by a solid line; data from the contralateral control eyes are plotted with a solid orange symbol and connected with a dashed line. The grey areas indicate test-retest variability, determined using the multiple baseline measurements with the one-way ANOVA method.[1]

References

1. Bland JM, Altman DG. Measurement error. BMJ 1996;312:1654.
