# Supplementary material for: Transplantation of Human Embryonic Stem Cell-Derived Retinal Pigment Epithelial Cells in Macular Degeneration
Source: Ophthalmology. 2018 Nov;125(11):1765–75. doi: 10.1016/j.ophtha.2018.04.037 (PMC6195794; doi:10.1016/j.ophtha.2018.04.037)
Supplement: Figure S6 [file mmc6.pdf]

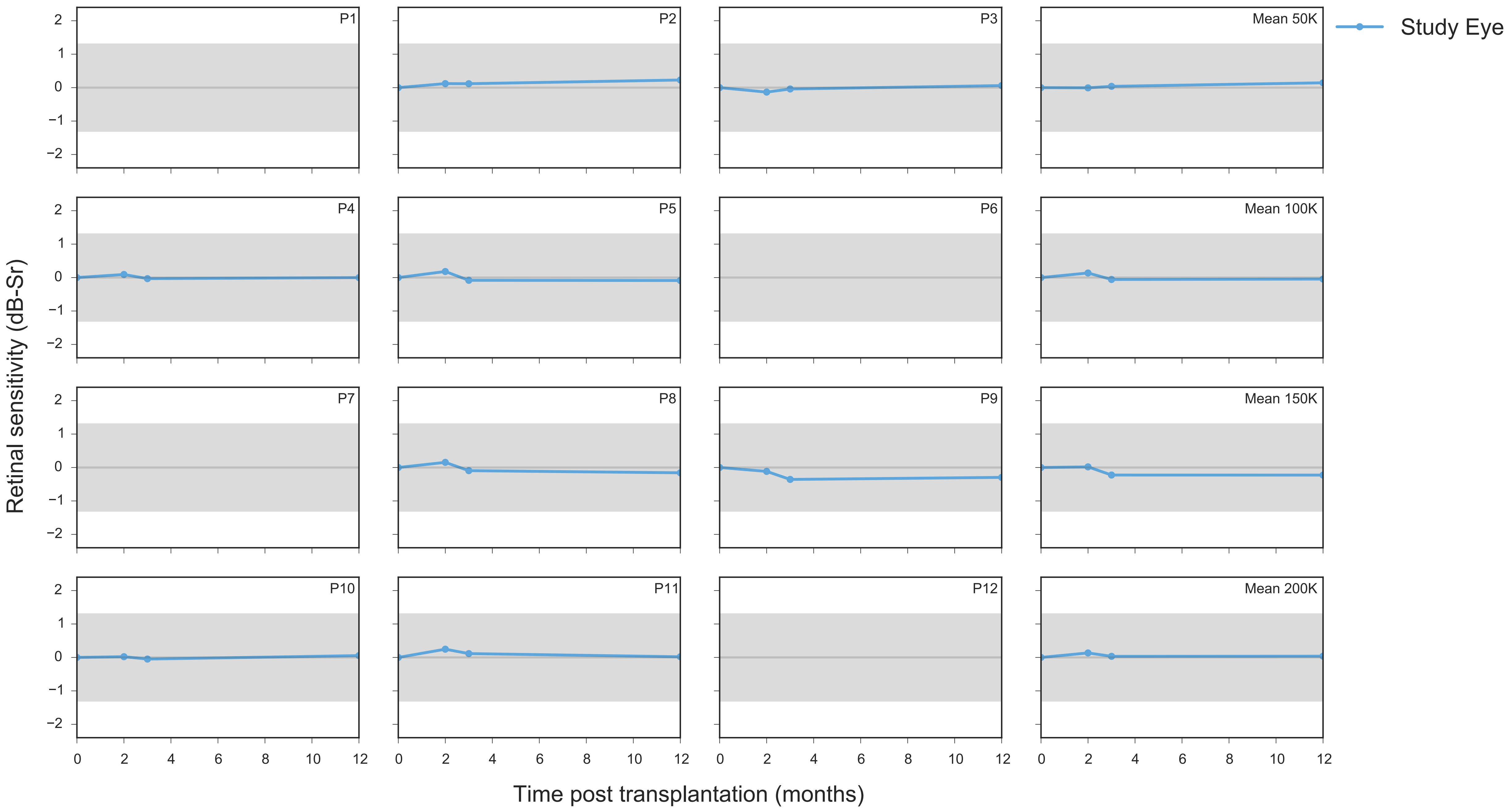

Supplementary Figure 6: Retinal sensitivity by high-density microperimetry

Volumetric retinal sensitivity in dB-sr plotted over 12 months for each participant. Sensitivities from each test loci at a predefined transition zone in the study eye was measured using high-density microperimetry grid, with each position separated from neighbouring ones by one degree. The sensitivities from all loci were interpolated to produce a 3-demensional topographic map and quantified using Visual Field Modelling and Analysis (VFMA) software developed by one of the authors.[2] Data from study eyes are plotted with a filled blue symbol connected by a solid line; data from the contralateral control eyes are plotted with a solid orange symbol and connected with a dashed line. The grey areas indicate test-retest variability, determined using the multiple baseline measurements with the one-way ANOVA method.[1] This assessment was unavailable or unreliable in 4 participants (P1, P6, P7 and P12).

References

1. Bland JM, Altman DG. Measurement error. BMJ 1996;312:1654.
2. Weleber RG, Smith TB, Peters D, et al. VFMA: Topographic Analysis of Sensitivity Data From Full-Field Static Perimetry. Transl Vis Sci Technol 2015;4:14.
