# Supplementary material for: Transplantation of Human Embryonic Stem Cell-Derived Retinal Pigment Epithelial Cells in Macular Degeneration
Source: Ophthalmology. 2018 Nov;125(11):1765–75. doi: 10.1016/j.ophtha.2018.04.037 (PMC6195794; doi:10.1016/j.ophtha.2018.04.037)
Supplement: Figure S7 [file mmc7.pdf]

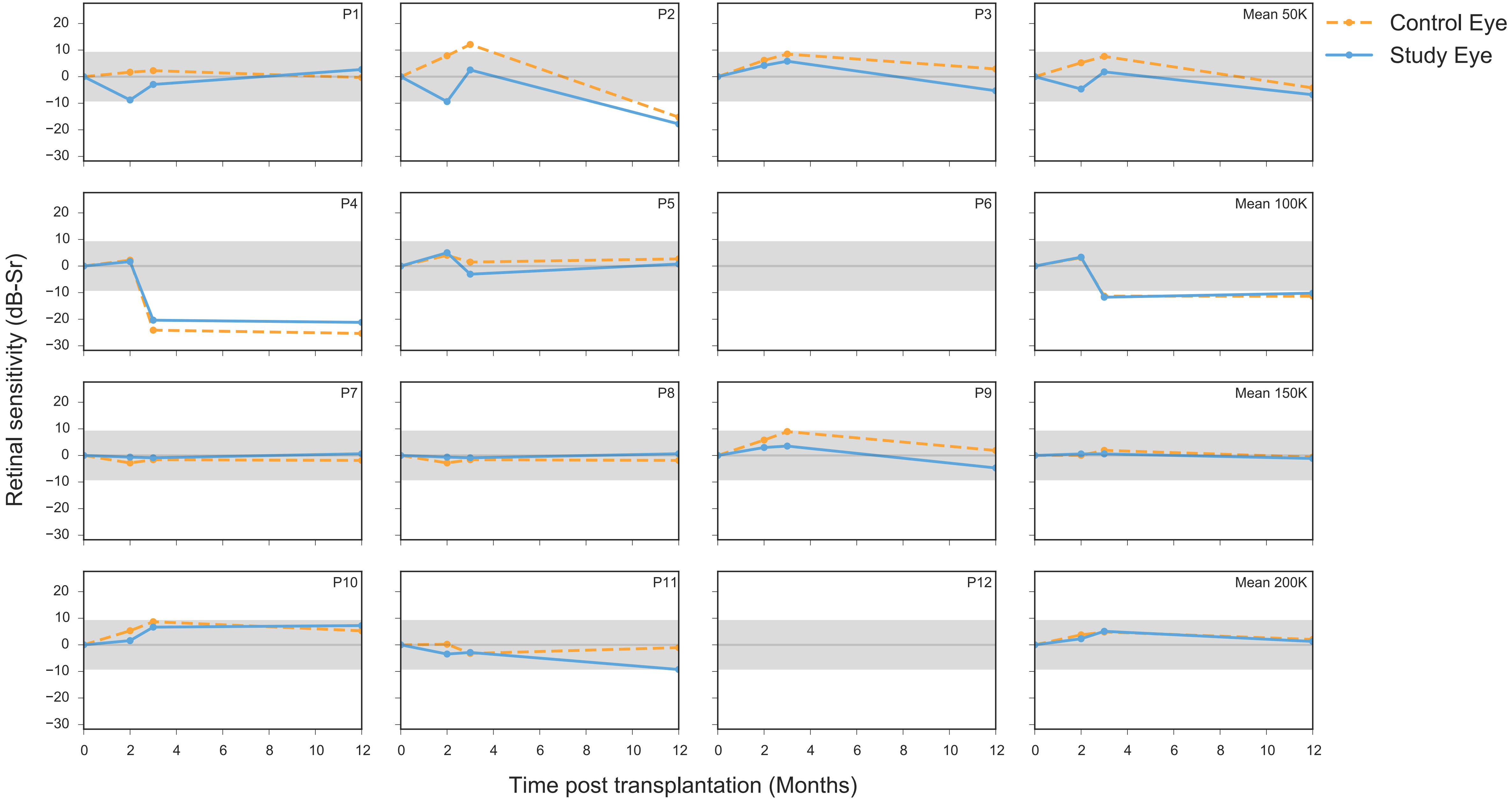

Supplementary Figure 7: Full-field perimetry (Octopus 900)

Volumetric retinal sensitivity in dB-sr plotted over 12 months for each participant. Sensitivities from each test locus were measured using Octopus 900 (Haag-Streit, Köniz, Switzerland) and a customised uni-ocular full-field static test grid German Adaptive Threshold Estimation (GATE) strategy[3,4], and stimulus size V. The sensitivities from all loci were interpolated to produce a 3-dimensional topographic map and quantified using Visual Field Modeling and Analysis (VFMA) software developed by one of the authors.[2] Data from study eyes are plotted with a filled blue symbol connected by a solid line; data from the contralateral control eyes are plotted with a solid orange symbol and connected with a dashed line. The grey areas indicate test-retest variability, determined using the multiple baseline measurements with the one-way ANOVA method.[1]

References

1. Bland JM, Altman DG. Measurement error. BMJ 1996;312:1654.
2. Weleber RG, Smith TB, Peters D, et al. VFMA: Topographic Analysis of Sensitivity Data From Full-Field Static Perimetry. Transl Vis Sci Technol 2015;4:14.
3. Schiefer U, Pascual JP, Edmunds B, et al. Comparison of the new perimetric GATE strategy with conventional full-threshold and SITA standard strategies. Invest Ophthalmol Vis Sci 2009;50:488-94.
4. Luithardt AF, Meisner C, Monhart M, Krapp E, Mast A, Schiefer U. Validation of a new static perimetric thresholding strategy (GATE). Br J Ophthalmol 2015;99:11-5.
