# Supplementary material for: Transplantation of Human Embryonic Stem Cell-Derived Retinal Pigment Epithelial Cells in Macular Degeneration
Source: Ophthalmology. 2018 Nov;125(11):1765–75. doi: 10.1016/j.ophtha.2018.04.037 (PMC6195794; doi:10.1016/j.ophtha.2018.04.037)
Supplement: Figure S8 [file mmc8.pdf]

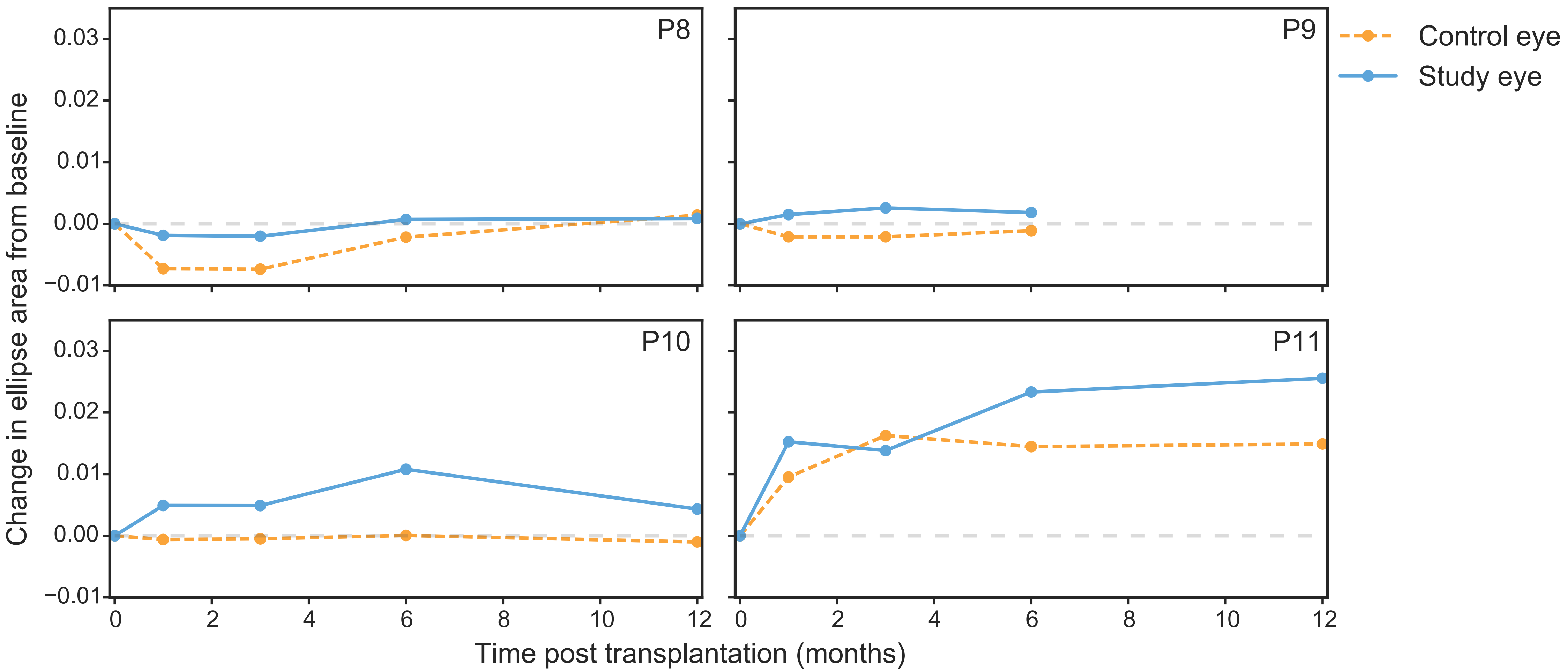

Supplementary Figure 8: Color discrimination

Summary graphs of Universal Colour Discrimination Test (UCDT) for participants P8 (A), P9 (B), P10 (C) and P10 (D). The y-axis represents the change in ellipse area from baseline. A positive value in the change of the ellipse area represents a worsening of color discrimination. The time point at which the UCDT was performed is indicated by the x-axis.
